# Can Thermal Nonreciprocity Help Radiative Cooling?

**DOI:** 10.34133/research.0563

**Published:** 2024-12-20

**Authors:** Run Hu, Zihe Chen, Sun-Kyung Kim

**Affiliations:** ^1^ School of Energy and Power Engineering, Huazhong University of Science and Technology, Wuhan 430074, China.; ^2^ Department of Applied Physics, Kyung Hee University, Yongin-si, Gyeonggi-do 17104, Republic of Korea.

## Abstract

Radiative cooling has witnessed substantial progress while its performance is constrained by the thermal reciprocal Kirchhoff’s law. Violating Kirchhoff’s law to pursue nonreciprocal radiative cooling seems promising; however, the energy conservation requirement and radiant flux integrated over the entire hemisphere make the nonreciprocal benefit insignificant. This commentary discusses the practical limits of nonreciprocal radiative cooling and points toward the future direction of directional radiative cooling.

Radiative cooling (RC), as a passive refrigeration method, has garnered increasing attention from both academia and industry with various materials such as photonic crystals [1], particle mixtures [2], wood [3], fibers [4], ceramics [5], and aerogels [6]. Laboratory-scale outdoor experiments worldwide frequently claim that the radiator temperature can be approximately 10 degrees below ambient temperature, depending on solar incidence, air temperature and humidity, weather, and landscape conditions. Researchers have focused on improving RC performance and devising specific applications by enhancing materials, structures, and manufacturing methods [7–10]. However, RC is confronted with a vital challenge; its performance is constrained by the intrinsic Kirchhoff’s law, which states that the spectral directional emissivity *ε*(*λ*, *θ*) is equal to the spectral directional absorptivity *α*(*λ*, *θ*). The primary requirement for RC spectra is to enhance solar reflectivity and midinfrared emissivity, especially in the atmospheric window (approximately 8 to 13 μm). Due to this constraint, high emissivity in the atmospheric window leads to high absorption of the midinfrared energy from the ambient environment, deteriorating RC performance. Despite substantial advancements in RC technologies with various materials, structures, and application scenarios, this inherent constraint remains unresolved.

The origin of Kirchhoff’s law lies in Lorentz reciprocity in Maxwell’s equations, which has been theoretically and experimentally violated with electromagnetic metamaterials [11]. Thus, breaking Kirchhoff’s law to achieve nonreciprocal thermal radiation has become a recent hot topic for better control of thermal radiation in terms of spectrum, directionality, and polarization in energy harvesting, infrared imaging, infrared camouflage, and thermal management. Feasible approaches include using magneto-optical materials [11], nonlinear optical materials [12], and spatiotemporal metamaterials [13]. By breaking Kirchhoff’s law, spectral absorptivity and emissivity can be modulated separately, providing a flexible and critical method for regulating thermal radiation and shedding light on various applications such as solar panels. For instance, using nonreciprocal photovoltaics can improve the solar energy harvesting limit to the Landsberg limit of 93.3%, compared to the reciprocal limit of 86.8% for conventional time-symmetric photovoltaics [14].

One might question whether nonreciprocal radiative cooling (NRC) can be achieved to improve RC performance. Initially, this might seem feasible and worthwhile. However, in this commentary, we aim to rectify this misconception through a comprehensive and deep thermodynamic analysis of the feasibility and necessity. The net cooling power of RC can be formulated as the difference between the outward radiated power (*P*_rad_) from the cooler and the inward absorbed power from the atmosphere (*P*_atm_), solar (*P*_solar_), and conduction and convection (*P*_cond+conv_), i.e., *P*_net_ = *P*_rad_ − *P*_atm_ − *P*_solar_ − *P*_cond+conv_. The outward radiative power is determined by the cooler’s temperature and spectral emissivity, which is central to RC performance. Without considering nonreciprocity, efforts have focused on achieving high emissivity in the atmospheric window while maintaining high reflectivity in the solar band to increase *P*_rad_ while decreasing *P*_solar_ as much as possible. Although considerable energy is absorbed from the atmosphere over a broad range of wavelengths due to the reciprocal Kirchhoff’s equivalence of emissivity and absorptivity, which negatively impacts *P*_net_, RC can be successful if *P*_net_ is positive.

Breaking Kirchhoff’s law is possible but must comply with the energy conservation law [15]. As shown in Figure A, consider an emitter *C* that undergoes radiative exchange through 2 radiation channels, labeled as *A* and *B*, with 2 separate blackbodies also marked as *A* and *B* respectively. Taking blackbody *A*(*B*) as an example, some of the radiation emitted by blackbody *A*(*B*) is absorbed by the emitter, denoted by the absorptivity *α_A_*(*α_B_*). The radiation that is not absorbed is reflected toward blackbody *B*(*A*), represented by the reflectivity *r*_*A*→*B*_(*r*_*B*→*A*_). The emitter also emits radiation toward blackbody *A*(*B*), described by the emissivity *ε_A_*(*ε_B_*). Considering the emitter at thermal equilibrium with 2 blackbodies *A* and *B*, due to the energy conservation requirement, *ε_A_* = 1 − *r*_*B*→*A*_ = *α_B_* and *α_A_* = 1 − *r*_*A*→*B*_ = *ε_B_*. Therefore, the difference between the emissivity *ε_A_* and absorptivity *α_A_* of the emitter toward blackbody *A* will be equal to the reflectivity difference between *A* → *B* and *B* → *A*, i.e., *ε_A_* − *α_A_* = *r*_*A*→*B*_ − *r*_*B*→*A*_. Such nonreciprocal reflectivity can be achieved but yields indispensable directionality. More generally, *ε*(±*θ*) = *α*(∓*θ*) is incident angle independent and always maintained, even if the unity difference |*ε*(*θ*) − *α*(*θ*)| = 1 is achieved [16]. In conventional RC design, only the spectral emissivity *ε*(*λ*) is considered. In comparison, for NRC design, both spectral and directional emissivity *ε*(*λ*, *θ*) should be considered. Therefore, to overcome atmospheric absorption in the atmospheric window from 8 to 13 μm, even if *ε*(*θ*) − *α*(*θ*) = 1 is achieved for perfect NRC performance in one-half of the hemisphere, the counterpart *α*(−*θ*) − *ε*(−*θ*) = 1 will default in the other half, as shown in Figure B, which is a key argument to bear in mind when designing NRC. Therefore, due to the requirement for directional emissivity and absorptivity *ε*(±*θ*) = *α*(∓*θ*), the energy absorbed from the atmospheric window is still partially present after integration over the entire hemisphere angles, thus weakening or even eliminating the nonreciprocal benefit on RC. In another work, we comprehensively evaluated the cooling performance of NRC with varying nonreciprocal wavelength bands, revealing the marginal benefits of NRC compared with reciprocal RC [17]. This is mainly because according to the second law of thermodynamics, the sum of absorption and emissivity is equal in all directions and polarizations [18]. Thus, the gain effect of thermal nonreciprocity on RC is contingent upon the weighting between the amount of atmospheric radiation absorbed by the radiator and the amount of external radiation from the radiator in the nonreciprocal band. Additionally, most thermal nonreciprocity is achieved within very limited incident angles and wavelengths, failing to cover the broadband for RC applications. Last but not the least, breaking Kirchhoff’s law for NRC currently requires giant magnetic fields [15], extremely low temperatures [11], complex spatiotemporal metamaterials [13], or optical nonlinearity [12], making experimental implementation challenging and not cost-effective, especially for the mass production of passive RC technologies.

**Figure.**
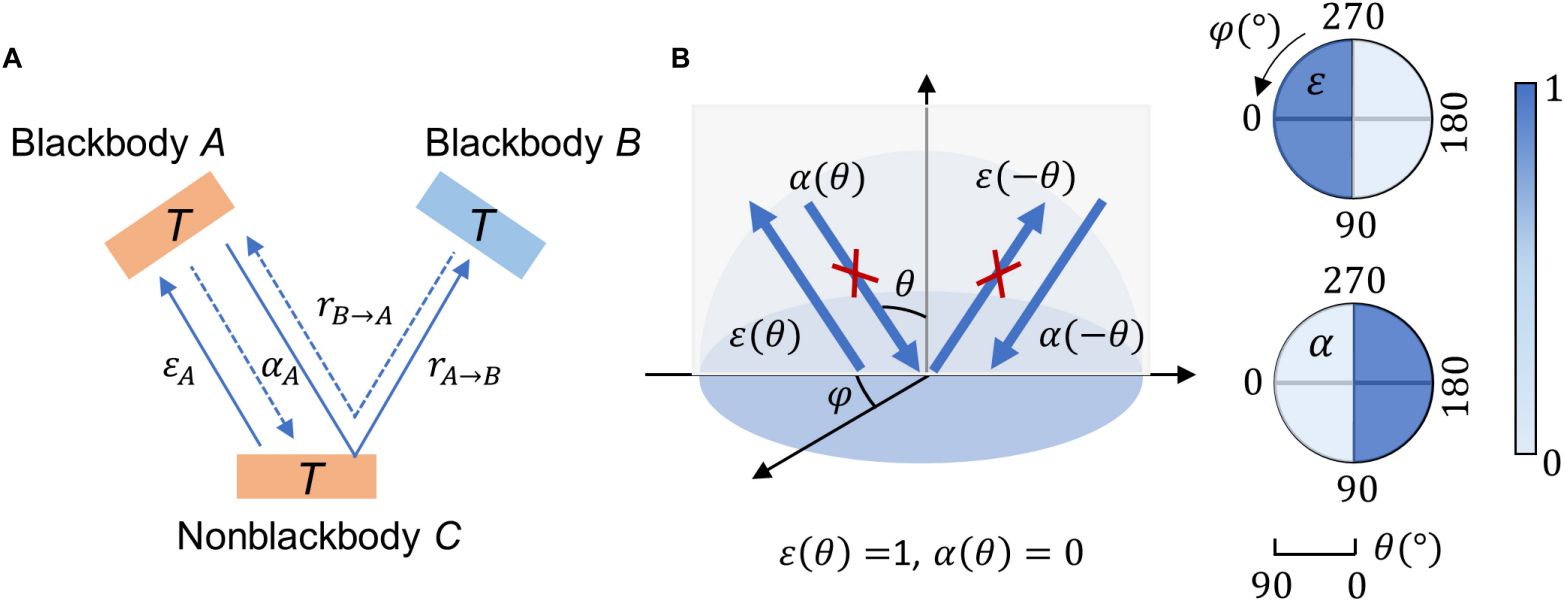
(A) Thermal nonreciprocity between emissivity and absorptivity can be achieved by modulating the nonreciprocal reflectivity, but energy conservation requires $\varepsilon \left(\pm \theta \right)=\alpha \left(\mp \theta \right)$. (B) The model of the ideal thermal nonreciprocity case: $\varepsilon \left(\theta \right)=1$ and $\alpha \left(\theta \right)=0$.

Instead of pursuing NRC, a promising alternative to enhancing RC performance is directional thermal radiation, which leverages spatial and spectral control of emissivity and absorptivity without breaking thermal reciprocity [19–21]. In our previous work, we designed and fabricated a hollow shell structure exploiting the Berreman mode and photon-tunneling mode for polarization-independent directional RC for head-mounted electronics [21]. Diffraction gratings, asymmetric geometries, epsilon-near-zero materials, 2-dimensional materials, and 3-dimensional structures can be further explored for directional RC [19–22]. Understanding these physical mechanisms and constraints, experimental implementation requirements, mass manufacturing feasibility, cost-effectiveness, and climate durability is critical to the integrative development of RC technologies to benefit human society and protect the earth in the short and long term.
